# Incorporating Ventricular Geometry into the Myocardial Work Index: A Proof-of-Concept Study

**DOI:** 10.3390/biomedicines14071606

**Published:** 2026-07-17

**Authors:** Kanza Awais, Konstantin Tripunovski, Andreja Černe Čercek, Tadeja Poropat Flerin, Borut Kirn

**Affiliations:** 1 Institute of Physiology, Faculty of Medicine, University of Ljubljana, 1000 Ljubljana, Slovenia; 2 Faculty of Medicine, University of Ljubljana, 1000 Ljubljana, Slovenia; konstantin.tripunovski@gmail.com; 3 Department of Cardiology, University Medical Centre Ljubljana, 1000 Ljubljana, Slovenia; 4 Department of Radiology, University Medical Centre Ljubljana, 1000 Ljubljana, Slovenia

**Keywords:** myocardial work index, one-fiber model, Laplace law, wall stress, stress–strain, ventricular geometry

## Abstract

**Background**: Mechanical work is traditionally defined by the pressure–volume loop, but its invasive nature limits routine clinical use. The myocardial work index (MWI) has emerged as a non-invasive alternative, combining strain and estimated pressure to assess cardiac performance. However, MWI does not account for ventricular geometry and treats the ventricle as a dimensionless chamber. According to Laplace’s law, wall stress and the true myocardial load depend on both pressure and ventricular geometry. Therefore, this study aims to develop and evaluate a geometry-informed myocardial work framework that provides a more physiologically representative estimate of mechanical energy expenditure. **Method**: In this proof-of-concept study, mechanical work was calculated using the one-fiber model of the left ventricle (LV) with Laplace-based geometric correction, integrating fiber stress over strain to derive the tension-adjusted myocardial work (TAMW) model. Strain was obtained from speckle tracking echocardiography along with pressure data while LV volumes and wall geometry were obtained from cardiac MRI. Two acute myocarditis patients with compact and dilated ventricles were analyzed, comparing cumulative and instantaneous work between MWI and TAMW. Performance gaps were quantified as the percentage difference in peak cumulative work. **Results**: Conventional MWI differed substantially between two cases (2576 vs. 1795 mmHg%, performance gap: 33.3%) whereas TAMW reduced this discrepancy to 7.5% (10,806 vs. 9789 mmHg%). TAMW also highlighted differences in temporal distribution of instantaneous work relative to MWI, reflecting the influence of ventricular geometry on contraction dynamics. **Conclusions**: TAMW incorporates the influence of geometry in the myocardial work framework revealing a more physiologically consistent reflection of myocardial effort across different ventricular geometries.

## 1. Introduction

The mechanical pumping work of the heart is classically defined as the area enclosed within the pressure–volume (PV) loop during a cardiac cycle. In particular, the systolic portion of PV loop reflects the energy required for force generation and blood ejection [1]. Although this approach provides a direct and physiologically meaningful assessment of cardiac work, its technical complexity and invasive nature have prevented its integration into routine clinical practice [2].

Myocardial work index (MWI) has recently emerged as a non-invasive alternative for the assessment of cardiac performance, offering an advantage over both invasive PV analysis and traditional indices of systolic function [3,4]. MWI is calculated as the time integral of pressure and strain rate obtained non-invasively. Myocardial strain is usually acquired with speckle tracking echocardiography (STE), while the left ventricular pressure curve is estimated by scaling a standardized reference curve to the patient’s brachial cuff pressure and aligning it with valvular events timings. Previous studies have demonstrated a reasonable agreement between MWI, myocardial metabolic measurements, and invasive PV-derived indices [5,6].

**Figure 1:**
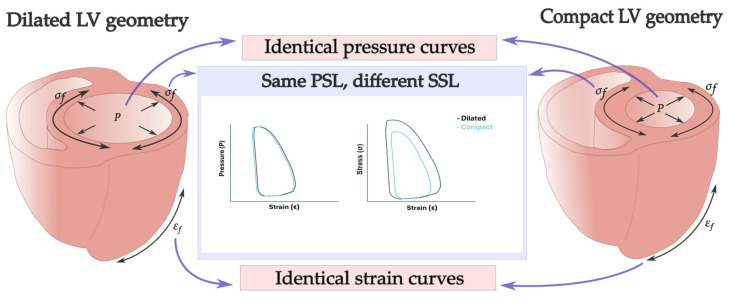
A visual representation of dilated and compact ventricles with identical pressure and strain curves, demonstrating similar pressure–strain loops (PSL) but different stress–strain loops (SSL) due to differences in ventricular geometry and resulting wall stress distribution.

Despite these advantages, significant theoretical and physical limitations remain when MWI is used as a substitute for PV loops, particularly when comparing ventricles with different geometries. Conventional MWI primarily reflects intraventricular pressure while remaining insensitive to ventricular size and wall thickness. As a result, it does not account for ventricular wall stress, which represents the true mechanical load experienced by cardiomyocytes [7]. Previous physiological studies have revealed a strong relationship between wall stress and myocardial oxygen consumption in both pressure and volume overload states, supporting wall stress as a key determinant of myocardial energetic demand [8]. According to the Laplace law, the wall stress depends not only on intraventricular pressure but also on ventricular geometry, particularly cavity radius and wall thickness [9]. Therefore, for the same systolic pressure, the actual workload is significantly higher in a dilated ventricle than in a compact one (Figure 1).

Furthermore, as a product of pressure and strain, MWI remains an index expressed in units of pressure. Although pressure is physically related to energy density, the MWI is not derived from the first-principles definitions of mechanical work incorporating volumetric components. Therefore, MWI may be more properly interpreted as a surrogate marker of myocardial energetic demand rather than a direct measure of myocardial work. This discrepancy can lead to conceptual ambiguity, particularly when derived indices like cardiac efficiency are interpreted in physiological terms [10].

In this proof-of-concept study, we propose a framework for myocardial work assessment that replaces the conventional pressure–strain loop with a geometry-adjusted stress–strain loop. By incorporating Laplace-law-based wall stress and geometric parameters, this approach accounts for geometric afterload that conventional MWI overlooks. As a methodological study, we demonstrate how this framework provides a more physiologically consistent reflection of myocardial effort across different ventricular geometries.

## 2. Methodology

Step 1: Core definition of Myocardial Work

The mechanical work per unit volume performed by the myocardium is defined as a time integral [11]:

(1) $$W=-\int \sigma_{f}\;(\frac{d\varepsilon_{n}}{dt^{\prime }})dt^{\prime }$$

where σ represents Cauchy fiber stress and $\varepsilon_{n}$ represents natural strain.

Step 2: Estimation of Fiber Stress via the One-Fiber Model

To estimate stress, we utilized a one-fiber model developed by T. Arts et al. [12], which treats the LV wall as a single fiber wrapped around the cavity with uniformly distributed Cauchy fiber stress ${(\sigma }_{f})$ and natural fiber strain $\left(\varepsilon_{f,n}\right)$. This model assumes a thick-walled, rotationally symmetric LV where active stress is dominant during systole. For such a shell, the relationship between wall stress and cavity pressure is described by the following expression [12]:

(2) $$\frac{P}{\sigma_{f}}=\frac{1}{3}\ln(1+\frac{V_{w}}{V_{lv}})$$

where P represents LV cavity pressure, $V_{lv}$ is volume of the LV cavity and $V_{w}$ is volume of the LV wall. For practical clinical applications, a linear approximation of this equation (accurate within ±5%) is utilized [11]:

(3) $$\frac{\sigma_{f}}{P}=1+3\frac{V_{lv}}{V_{w}}$$

This relationship ensures that all subsequent derivations intrinsically account for the effects of Laplace’s law and variations in ventricular geometry.

Step 3: Derivation of Natural Fiber Strain

To derive strain, we adapted the method used by Arts et al. [12] to align with standard STE strain convention regarding values at the onset of systole. Accordingly, natural strain is defined as:

(4) $$\varepsilon_{n}=\frac{1}{3}\ln\left(\frac{1+3+\frac{V_{lv}}{V_{w}}}{1+3+\frac{EDV}{V_{w}}}\right)$$

Step 4: Integration and final TAMW formulation

**Figure 2:**
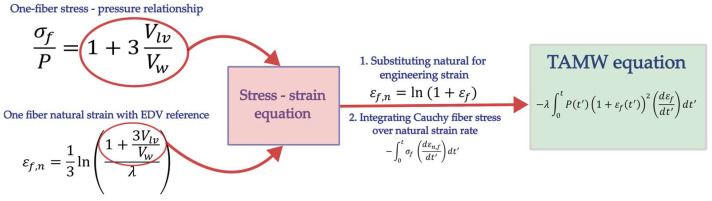
Step-by-step workflow of the TAMW index calculation.

Then, by combining Equations (3) and (4), we established a comprehensive stress-strain relationship for the myocardium during systole. Subsequently, by integrating the Cauchy stress according to Equation (1), and converting natural strain $\left(\varepsilon_{n}\right)$ to standard STE engineering strain $\left(\varepsilon_{f}\right)$ using their relation $\varepsilon_{n}=\ln(1+\varepsilon_{f})$, we derived the final expression for myocardial work per unit volume. This yielded the tension-adjusted myocardial work (TAMW) as a function of time:

(5) $$TAMW\;(t)=-\lambda \int_{0}^{t}P(t^{\prime }){\left(1+\varepsilon_{f}(t^{\prime })\right)}^{2}(\frac{d\varepsilon_{f}}{dt^{\prime }})dt^{\prime }$$

where $\lambda =1+3\frac{EDV}{V_{w}}$ represents a geometric scaling factor with EDV denoting end diastolic volume. Schematic workflow of the TAMW derivation can be seen in Figure 2.

## 3. Patient Data

To demonstrate the influence of our proposed model, we selected two patients of acute myocarditis (AM) with two distinct geometries: Case 1 with a compact LV, i.e., normal LV geometry and Case 2 with a dilated LV. In both cases, we calculated cumulative and instantaneous work using conventional MWI and TAMW utilizing Equation (5). Cumulative work was obtained during systole using concepts explained by Russell et al. [5,6]. Systole is defined as time interval from mitral valve closure (MVC) to mitral valve opening (MVO). Instantaneous work was computed as the time derivative of cumulative work. For both models, a patient-specific LV pressure curve was generated using the same methodological approach in which individual BP values and valvular event timing were applied to a standardized reference pressure template [5]. Then, we calculated the performance gap, which signifies the relative difference in peak cumulative work between two cases, calculated as the absolute difference divided by their mean and expressed as a percentage.

The patient’s diagnosis of AM was based on elevated troponin I levels, electrocardiographic changes suggestive of AM, cardiac magnetic resonance (CMR) findings consistent with AM, and absence of coronary artery disease confirmed by coronary angiography. This study was conducted in accordance with institutional ethical standards for retrospective analysis of anonymized imaging data and was approved by the local ethics committee.

###  3.1. Strain and MW Analysis 

**Figure 3:**
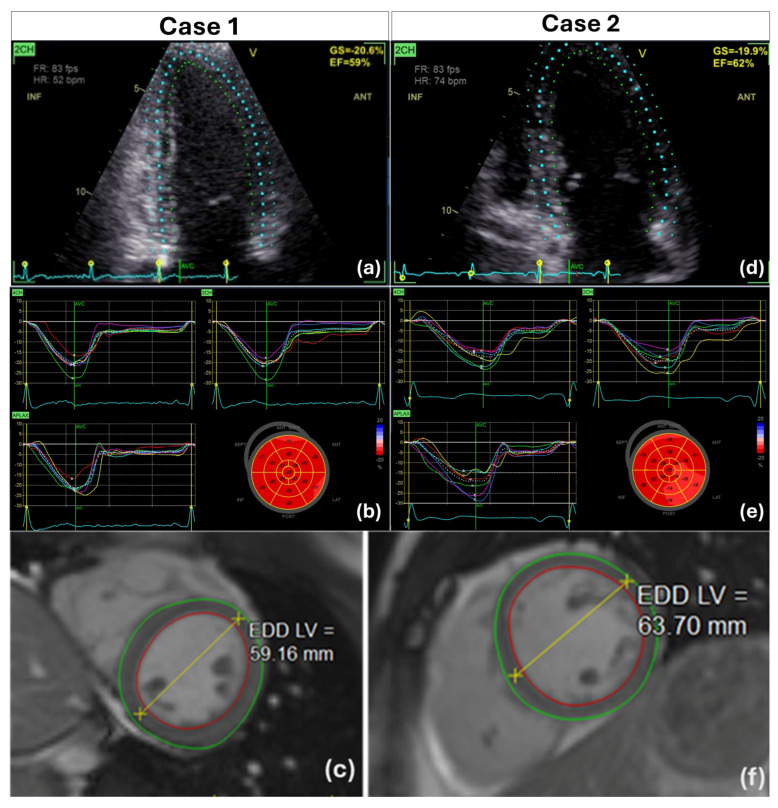
A comprehensive overview of strain and volumetric parameter extraction from STE and CMR-based Medis-suite (Q mass). STE Endocardial and epicardial contours of LV (dotted blue lines) are shown in the apical two-chamber view for Case 1 (**a**) and Case 2 (**d**). Strain curves from the apical 4-, 3-, and 2-chamber views, along with corresponding bull’s eye plots of strain are presented for Case 1 (**b**) and Case 2 (**e**). Cine short-axis CMR images of LV at end-diastole are shown for Case 1 (**c**), exhibiting compact LV geometry (EDD = 59.2 mm) and Case 2 (**f**), demonstrating a dilated LV geometry (EDD = 63.7 mm). Endocardial (red) and epicardial (green) contours were used to derive EDV and *V_w_*, forming the geometric basis for TAMW calculation.

In both patients, strain and conventional MW index were acquired using EchoPAC strain analysis software (Version 206, GE Healthcare, Horten, Norway) with automated functional imaging (AFI). LV borders were automatically traced and subsequently reviewed with manual correction applied where necessary as shown in Figure 3a (Case 1) and Figure 3d (Case 2). Strain analysis was referenced to end-diastole defined by the R-wave on electrocardiography (ECG). Segmental strains from 18 LV segments were averaged to calculate global longitudinal strain (GLS), as demonstrated in Figure 3b,e. Valvular event timings were determined from the apical three-chamber view to align temporal MW calculations. Brachial artery blood pressure (BP) values were used to generate individualized LV pressure–strain loops for MW calculation.

### 3.2. CMR-Derived Geometric Parameters

Volumetric and geometric parameters were obtained from CMR using QMass software (Version no. 2025.1.14.6, Medis Medical Imaging Systems, Leiden, The Netherlands). Endocardial and epicardial borders were automatically traced at end-diastole and end-systole that were manually corrected where needed, shown in Figure 3c,f. Parameters extracted included end-diastolic volume (EDV), end-systolic volume (ESV), LV mass, LV wall volume (*V_W_*), and end-diastolic LV diameter (EDD LV). 

All these echocardiographic and CMR volumetric parameters were then imported to MATLAB (R2024a, MathWorks, Natick, MA, USA) to calculate TAMW.

### 3.3. Sensitivity Analysis

To evaluate the robustness of our TAMW framework, a one-way sensitivity analysis was performed by perturbing the *V_w_* and EDV by ±10% while keeping other parameters constant. The influence of this perturbation was tested on Equation (5) to determine the stability of our model under physiologically plausible variations in ventricular geometry and to ensure the identified performance gap between two cases were not driven by measurement uncertainty.

## 4. Results

All acquired clinical, echocardiographic, and CMR-derived variables are summarized in Table 1.

Case 1 was characterized by elevated BP and normal EDV, representing a pressure-dominant loading condition. In contrast, Case 2 exhibited normal BP but increased EDV, reflecting a volume- and geometry-dominant loading state. In a simplified physiological interpretation, both cases would be expected to demonstrate similar myocardial work, driven, however, by different hemodynamic mechanisms.

**Figure 4:**
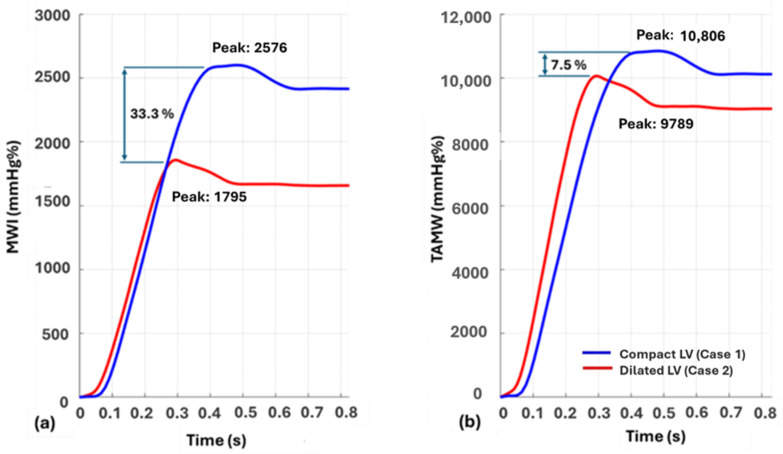
Global cumulative work curves of Case 1 (blue) and Case 2 (red) acquired by MWI (**a**) with performance gap of 33.3% and TAMW with 7.5% of disparity (**b**).

Despite these similar conceptual expectations, conventional MWI demonstrated substantial difference between the two cases. Case 1 reached MWI of 2576 mmHg%, compared with 1795 mmHg% in Case 2, corresponding to a relative performance gap of 33.3%. However, when myocardial work was recalculated using the TAMW model, the disparity between two cases was reduced to 7.5% with values of 10,806 mmHg% in Case 1 and 9789 mmHg% in Case 2. Although TAMW remained higher in Case 1, incorporation of ventricular geometry substantially modified the comparative workload profiles as seen in Figure 4.

**Figure 5:**
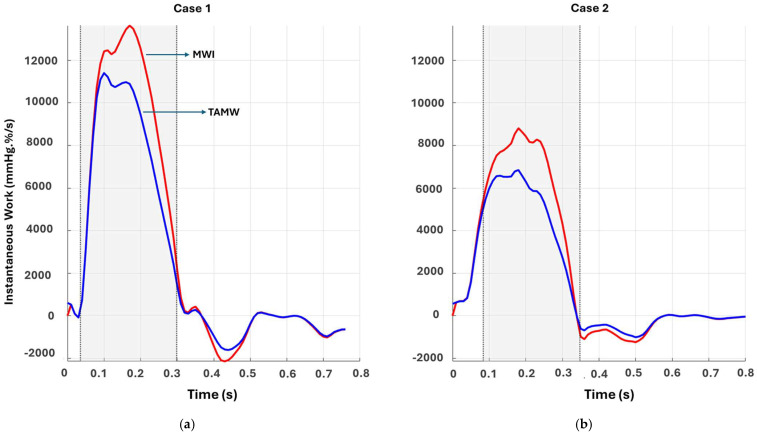
Instantaneous work curves from Case 1 (**a**) and Case 2 (**b**) using MWI (red) and TAMW (blue) with shaded areas representing systolic period. For better visualization and comparison, the TAMW scaling factor λ was set to 1.

Beyond magnitude differences, TAMW also revealed marked differences in temporal distribution of instantaneous work. This effect became more noticeable when the scaling factor (λ) in the TAMW was set to 1 for visualization purposes, allowing a direct comparison of instantaneous work curves (Figure 5). Under these conditions, Case 1 exhibited a relative leftward shift in the slope of instantaneous TAMW compared with MWI, emphasizing a greater contribution of early systolic mechanics when accounting for ventricular geometry.

To assess robustness, a one-way sensitivity analysis was performed by perturbing *V_w_* and EDV by ±10%. In Case 1, where the baseline TAMW was 10,806 mmHg%, perturbations in *V_w_* resulted in a range of 10,016 to 11,772 mmHg% while variations in EDV resulted in range of 9937 to 11,676 mmHg%. Similarly, in Case 2, the baseline of 9789 mmHg% ranged from 9027 to 10,720 mmHg% under *V_w_* perturbations and 8951 to 10,627 mmHg% under EDV perturbations. Despite these perturbations, the relative discrepancy between the two cases remained consistently smaller than the observed 33.3% performance gap observed in MWI, demonstrating that the TAMW framework is algorithmically stable and remain robust against standard clinical measurement variability.

## 5. Discussion

The primary finding of our pilot study is that conventional MWI might substantially underestimate the energetic burden of the dilated heart by neglecting the influence of geometry, specifically radius and wall thickness. In our dataset, baseline MWI values obtained for Case 1 (2576 mmHg%) and Case 2 (1795 mmHg%) were consistent with established clinical patterns with Case 1 reflecting a hyperdynamic profile [13], while Case 2 falls within the lower bounds of normal MWI defined by NORRE study [14]. Despite these expected patterns, the observed 33.3% performance gap in MWI between these two AM phenotypes appears to be influenced in part by geometric differences rather than pressure alone. When ventricular geometry was incorporated through the Laplace-based geometric scaling factor, the mechanical workload between the two cases became more comparable (7.5% disparity), suggesting that the dilated phenotype might experience a higher effective mechanical burden per unit of strain to overcome its geometric afterload than suggested by conventional MWI.

The derivation of TAMW from the first principles allows expression of myocardial work in energy units (mJ/mL), rather than traditional pressure–strain units (mmHg%). In the present pilot dataset, Case 1 and Case 2 corresponded to 14.4 mJ/mL and 13.1 mJ/mL respectively. However, for consistency with established myocardial work methodology [5], results were primarily reported in mmHg% to facilitate direct comparison with conventional MWI. While we acknowledge that the use of non-invasive brachial pressure estimates remains a limitation common to all MW indices, the transition to energy-based units allows for a more physiologically grounded representation of the estimated biomechanical energy expenditure underlying myocardial contraction, rather than a direct measurement of true mechanical work. This approach also allows us to isolate the contribution of the Laplace-based geometric scaling factor (λ) and the dynamic (1 + ε)^2^ term, highlighting their role in modulating estimated mechanical work in dilated phenotypes while maintaining a familiar clinical scale. For future applications, we recommend reporting TAMW in mJ/mL to reflect physiologically grounded energy estimates.

We also noticed temporal redistribution of MW specifically in Case 1, with a leftward shift identified in the instantaneous work curve by TAMW as compared to MWI. This behavior is fundamentally influenced by the dynamic (1 + ε)^2^ term in Equation (5) which amplifies the contribution of early systolic shortening in compact ventricle. In this setting, rapid changes in ventricular deformation allow myocardial work to peak earlier relative to peak pressure. This front-loading effect is consistent with biomechanical models proposed by T. Arts et al. who demonstrated that the relationship between fiber stress and cavity pressure is dynamically modulated by the instantaneous volume to wall volume ratio [12]. Conversely, Case 2 exhibited a more attenuated temporal shift, suggesting that ventricular dilation may dampen this redistribution due to dominant influence of geometry on overall mechanics.

## 6. The Pressure–Geometry Discrepancy

Previous studies revealed that increase in MWI is a direct result of elevated afterload, often noticed in hypertensive remodeling [15,16]. Our data supports this for Case 1 where BP is elevated (150/100 mmHg) which resulted in a high MWI of 2576 mmHg%. However, Case 2 presented a different scenario. Despite having much lower BP, i.e., 107/65 mmHg, its TAMW was close to Case 1 (9789 vs. 10,806 mmHg%). This finding does not contradict previous studies correlating higher afterload with increased MW; rather, it identifies a scenario where the primary source of afterload shifts from pressure to geometry. According to the law of Laplace, as the ventricle dilates and the wall thins, the myocardium must generate substantially greater wall tension to produce the same pressure. Standard myocardial work fails to capture this burden since it lacks a geometric scaling factor, leading to underestimation of the true energy expenditure in dilated ventricles.

Current non-invasive myocardial work algorithms rely primarily on BP as a surrogate for afterload, as highlighted in the NORRE study [14]. While practical, this approach does not incorporate structural ventricular remodeling. According to the classic law of Laplace established by Grossman et al. [17] and the computational model of Lumens et al. [18], the heart’s true energy demand is driven by wall stress.

By ignoring these geometric variations, conventional MWI effectively treats Case 1 and Case 2 as having the same internal afterload per mmHg of pressure. By including λ, TAMW integrates the findings of both cases under a unified biomechanical principle: MW is a product of both hemodynamic pressure and geometry. Although the classic thin-shell law of Laplace relies on simplifying assumptions that do not fully capture the complex structure of the thick-walled LV, our model overcomes these limitations by adopting the concepts proposed by T. Arts et al. [12]. This approach establishes the relationship between Cauchy fiber stress and LV pressure within a thick-walled, rotationally symmetric ventricle, allowing derivation of a thermodynamically consistent formulation of MW. By incorporating myocardial mass via CMR to define *Vw*, the model moves beyond the simplified “thin bubble” assumption and provides a more physiologically realistic representation of mechanical load distributed throughout the actual myocardial tissue, accounting for the physical volume in which work is performed.

In doing so, TAMW captures aspects of myocardial energy consumption and metabolic demands [19] that conventional MWI may fail to capture. Recent studies have highlighted that conventional MWI may become less reliable in patients with extensive remodeling of LV [20], as the standard PS loops do not explicitly account for wall stress, wall thickness and ventricular curvature. By incorporating λ and dynamic (1 + ε)^2^, TAMW specifically integrates these geometric influences into MW estimation. This allows for a more physiologically consistent assessment of myocardial performance in dilated phenotypes where the energetic cost is increasingly governed by ventricular radius and wall thinning rather than brachial pressure alone.

## 7. Integrating Prognostic Markers: The Advantage of TAMW

An important strength of TAMW is that it is derived entirely from routinely acquired echocardiographic and CMR parameters. Most notably, it incorporates EDV, which itself is an established prognostic marker in diseases like AM since increase in baseline EDV has been associated with a higher risk of adverse remodeling, impaired functional recovery and heart failure [21,22].

By directly involving EDV into MW calculation, the TAMW evaluates myocardial efforts within the structural context of LV. Rather than assessing pressure and strain alone, it interprets these parameters relative to cavity size and wall thickness. In Case 2, even though MWI suggested lower work, the markedly elevated EDV indicated substantial geometric burden. By accounting for the increased wall stress inherent to a dilated LV, the TAMW identified a myocardium operating under considerable mechanical load despite relatively normal pressure—derived work indices.

Beyond its prognostic integration, TAMW may also have potential clinical utility in refining patient management strategies. Conventional MWI can provide false reassurance in patients with ventricular dilation and low-to-normal systemic blood pressure because it does not account for increased wall tension required to maintain stroke volume in larger cavities. By incorporating ventricular geometry, TAMW may better identify patients with elevated wall stress—related energy expenditure despite apparently normal MWI values. In addition, TAMW could serve as a sensitive marker for monitoring reverse remodeling, as reduction in ventricular volume would be expected to lower TAMW even when systemic blood pressure remains stable.

## 8. Limitations

This study represents a proof-of-concept analysis based on two representative phenotypes of AM. While the results highlight a biomechanical discrepancy between standard MWI and TAMW, they do not represent a generalized clinical cohort; large scale studies are needed to establish the definitive diagnostic thresholds.

An additional limitation is the absence of validation against an invasive reference standard such as PV-loop analysis. Consequently, the reduction in the performance gap from 33.3% to 7.5% should be considered an improvement in physiological consistency rather than absolute diagnostic proof. Nevertheless, the TAMW framework is theoretically grounded in established biomechanical models [12] and supported by classic metabolic studies, such as Stauer et al. [8] which identified wall stress as a strong determinant of myocardial oxygen consumption compared to cavity pressure alone, particularly in ventricular remodeling.

Furthermore, non-invasive BP values were used as a surrogate for LV pressure, representing a limitation shared by current commercially available software. Finally, myocardial fiber deformation is highly dependent on fiber orientation and is not purely longitudinal. Therefore, the use of GLS as a surrogate for fiber strain introduces an inherent approximation in the estimation of MW, consistent with limitations present in currently established MW frameworks [5,23].

## 9. Conclusions

The current most-used approximation of myocardial work—MWI—underestimates the energetic burden of dilated ventricles because it ignores the geometric afterload imposed by an increase in ventricular radius and wall thinning. In contrast, the proposed TAMW incorporates ventricular geometry into MW estimation, providing a more physiologically grounded representation of the mechanical effort required for a dilated heart to maintain its cardiac function.

## Figures and Tables

**Table 1:** Summary of patient characteristics. Abbreviations: GLS—global longitudinal strain, BP—blood pressure, EDV—end diastolic volume, ESV—end systolic volume, *V_w_*—wall volume, MWI—myocardial work index, TAMW—tension adjusted myocardial work.

Variables	Case 1	Case 2
Age	27	31
Gender	Male	Male
Geometry	Compact	Dilated
GLS (%)	−20.1	−21.7
BP (mmHg)	150/100	107/65
EDV (mL)	115	218
ESV (mL)	46	83
*V_w_* (mL)	83.8	110
λ	5.12	6.95
MWI (mmHg%)	2576	1795
TAMW (mmHg%)	10,806	9789

## Data Availability

All data supporting the findings of this study are included in the article; any additional inquiries can be directed to the corresponding author.
